# Use of satellite imagery in constructing a household GIS database for health studies in Karachi, Pakistan

**DOI:** 10.1186/1476-072X-3-20

**Published:** 2004-09-28

**Authors:** Mohammad Ali, Shahid Rasool, Jin-Kyung Park, Shamoon Saeed, Rion Leon Ochiai, Qamaruddin Nizami, Camilo J Acosta, Zulfiqar Bhutta

**Affiliations:** 1 International Vaccine Institute, SNU Research Park, San 4–8 Bongcheon-7 dong, Kwanak-gu, Seoul, Korea; 2 Aga Khan University, Pediatric Department, Karachi, Pakistan; 3 Techno-Consult International, Karachi, Pakistan

## Abstract

**Background:**

Household-level geographic information systems (GIS) database are usually constructed using the geographic positioning system (GPS). In some research settings, GPS receivers may fail to capture accurate readings due to structural barriers such as tall buildings. We faced this problem when constructing a household GIS database for research sites in Karachi, Pakistan because the sites are comprised of congested groups of multi-storied building and narrow lanes. In order to overcome this problem, we used high resolution satellite imagery (IKONOS) to extract relevant geographic information.

**Results:**

The use of IKONOS satellite imagery allowed us to construct an accurate household GIS database, which included the size and orientation of the houses. The GIS database was then merged with health data, and spatial analysis of health was possible.

**Conclusions:**

The methodological issues introduced in this paper provide solutions to the technical barriers in constructing household GIS database in a heavily populated urban setting.

## Introduction

Geographic data are increasingly being employed in health studies [1]. By studying disease patterns in space, we can understand the relationships between socioecological exposure and illness [2,3]. Such understanding may help the formulation of need based healthcare systems and health intervention programs. Geographic methods provide a wide spectrum of geographic scales from local to global for analyzing health and health-related data. Regional variation in disease incidence be attributed to regional or global differences in ecological or socio-environmental phenomena [4]. Local-level geographic variation of disease obtained from fine resolution geographic data can provide clues about the spatial variability [5], and may pinpoint areas where health interventions are needed.

One way to facilitate the measurement of local variation in health outcomes is to create household-level geographic information systems (GIS) database. Household locations can be captured by using GPS (global positioning system) receivers [6,7]. However precise geographic data on households are an absolute requirement for critical examination of local variation of the disease and its association with the environment [8]. A large variety of GPS receivers are available in the market and different GPS receivers provide different levels of accuracy. A low cost receiver can capture data with an accuracy of 5 to 10 meters provided that they are configured properly and the satellites have good alignment at the time the data are collected [9]. The alignment of the GPS satellite constellation at a particular time can be measured using GPS trip planning software. It is essential that the GPS receiver has a clear "view" of at least four GPS satellites which can be obstructed by large structures such as buildings or mountains. In congested urban settings, collecting household locations in narrow lanes using the GPS can be challenging.

Faced by such challenges we explored satellite imagery in order to acquire household GIS data in urban slums in Karachi, Pakistan. This paper describes the methods used to construct the household GIS database and the technical barriers one might encounter during the construction of a database.

## The household geographic information systems project

Geographic studies have been considered as one of the research disciplines of large Vi (antigen) typhoid vaccine effectiveness trials as well as typhoid disease burden studies [10]. The Vi typhoid vaccine provides a comparable degree of protection to the whole-cell type but with less severe side effects. Only one dose is required for a course of vaccination. The studies are part of the Diseases of the Most Impoverished (DOMI) program, a multi-country, multi-disciplinary health research program aimed to accelerate the development and introduction of a new generation vaccines against cholera, typhoid fever, and shigellosis in several Asian countries. The program involves a number of parallel activities including epidemiological studies, social science studies, and vaccine technology transfer. The local collaborator of the household GIS project in Karachi is the Pediatric Department of the Aga Khan University Hospital, Karachi, Pakistan. Technical support for the project was provided by Techno-Consult International, Karachi, Pakistan.

**Figure 1:**
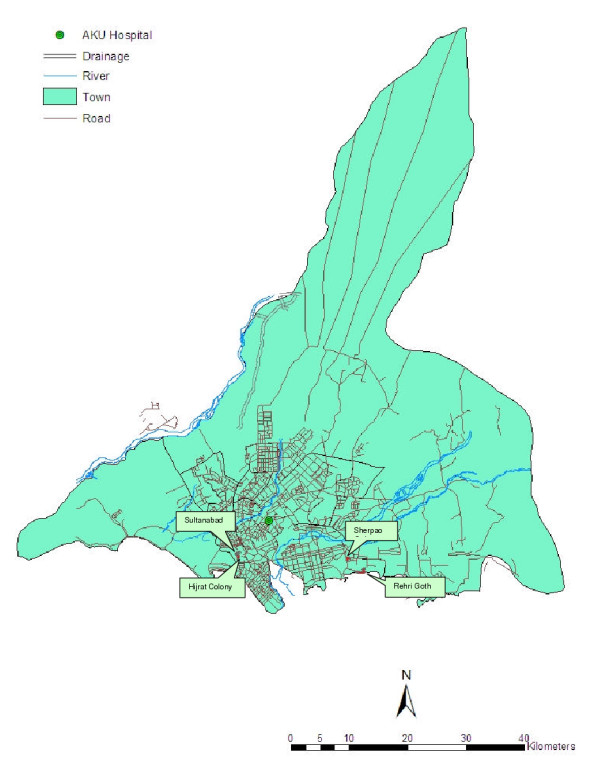
**The study sites in Karachi, Pakistan. **The geographic position of the four study sites along with other geographic characteristics of Karachi are shown in the map.

The aim of the project was to construct a spatial database that includes household locations, study area boundary with administrative units, and other geographic features such as hospitals/clinics, schools, mosques, roads, lanes, and water bodies. The project area included four Karachi slums including Sultanabad, Hijrat Colony, Rehri Goth, and Sherpao Colony (Figure 1). Sultanabad and Hijrat Colony are adjacent areas near the port of Karachi, and Rehri Goth and Sherpao Colony are about two kilometers apart located 20 kilometers South East of Karachi. In 2002, a census was conducted in the four slums to enumerate the study population. The list of households and their addresses were obtained from the population database.

## The base map

A commercially available map of Karachi was used as the base map for this GIS project. The base map was georeferenced with four identifiable landmarks using handheld GPS receivers with accuracy of approximately five meters. This accuracy was considered sufficient to identify the study areas, to order satellite imagery, and to conduct subsequent ground surveys. After georeferencing the map, the main geographic features such as roads, hospitals/healthcare centres, and other city landmarks were digitized and incorporated into the baseline GIS database (Figure 1).

## The satellite imagery

**Figure 2:**
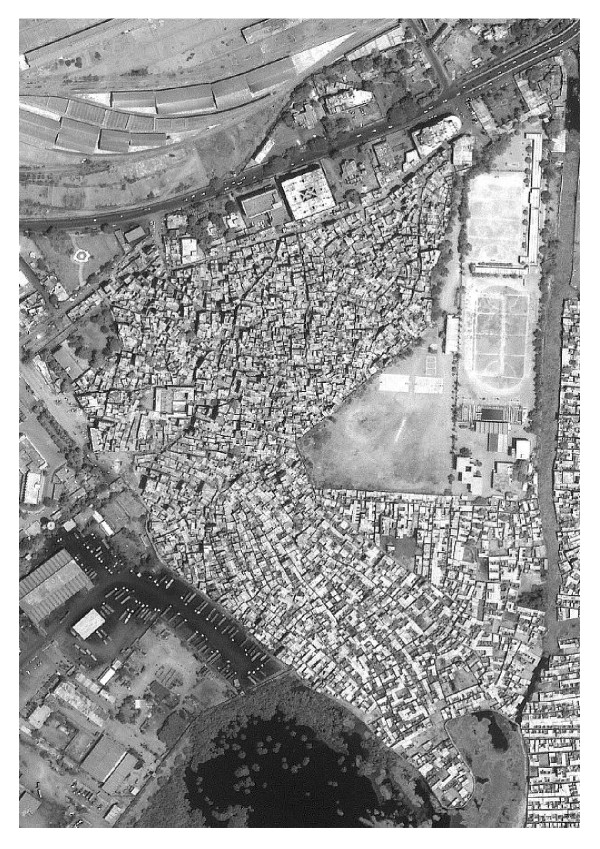
**IKONOS image of Sultanabad, Karachi, Pakistan. **The landscape of the Sultanabad study area obtained from IKONOS satellite imagery.

Satellite imagery is available at different spatial, temporal, and spectral resolutions [11]. Different sensors capture images of the earth surface in different spectral resolutions, which allow different surface features to be differentiated. At the time of the project, the highest resolution commercially available satellite imagery was the one-meter panchromatic from the IKONOS (Space Imaging, Inc) satellite. We acquired a panchromatic IKONOS image (Figure 2) for two study slums, Sultanabad and Hijrat. We found that the one-meter panchromatic imagery was not helpful for separating lanes from building and mud roofs from open ground. We therefore acquired the four-meter multispectral IKONOS imagery for the two other study slums Rehri Goth and Sherpao Colony where many houses are made of mud. The result was more appropriate for our purposes even with the loss of spatial resolution.

## Image processing and georeferencing

The satellite images were enhanced using an image processing software package (ERDAS Imagine, Atlanta, USA) to facilitate the digitization of house parcel boundaries. High precision, dual frequency GPS units (Trimble 4000 ssi) were used to capture data at several identifiable points on the images to be used as ground control points (GCPs). To transfer images into a GIS database, it must be geometrically rectified to a known coordinate system on the basis of a number of GCPs [12]. Most of the GCPs were selected from the periphery of the study area so that possible errors would converge towards middle of the area. After locating GCPs on the satellite image and identifying them on the ground, GPS readings were obtained at centimeter level accuracy. The GPS data were collected in the WGS-84 (World Geodatic Systems-84) datum in the latitude/longitude system and were subsequently transformed into the Universal Transverse Mercator (UTM) Zone 42-North system. The GCP coordinates within the UTM projection were then integrated with the satellite images using the ERDAS Imagine software for georeferencing. The resultant root mean square (RMS) errors were approximately two meters, which was considered sufficiently accurate for the purpose of constructing the GIS database.

## Digitization of house parcels

**Figure 3:**
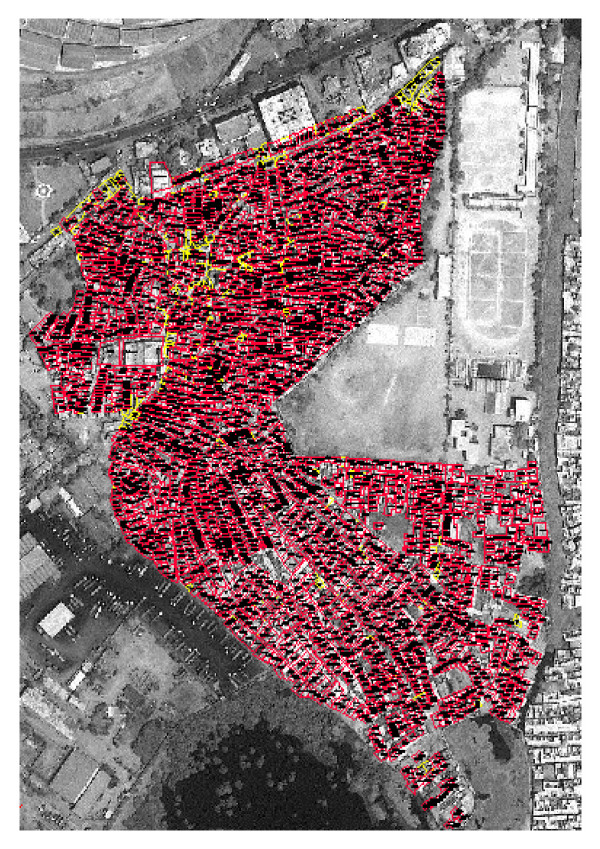
**House parcels added to the satellite image, Sultanabad, Karachi, Pakistan. **The house parcels drawn using AutoCAD superimposed on to the IKONOS satellite imagery.

After georeferencing the images were resampled, converted into JPEG files, and were imported in AutoCAD Version 14 (Cadopolis.com Inc., Canada). These processes allowed the parcel boundaries to be delineated through heads-up digitizing. The resampled satellite imagery was inserted as backdrop in AutoCAD. The image was aligned to correct its scale, translation and rotation by using two GCPs located at the corners of the image. After the alignment of the raster images in AutoCAD the house parcels were digitized. One image was used on multiple workstations to digitize different portions of the image, which were subsequently merged to form a complete area map of house parcels (Figure 3).

## Ground survey

**Figure 4:**
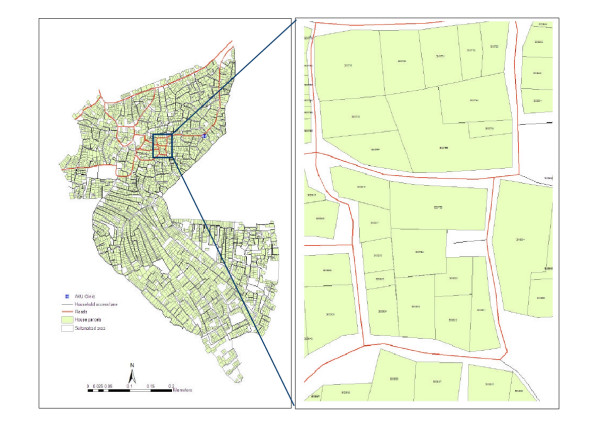
**The address ID is used to link house parcels to population database, Sultanabad, Karachi. **The unique census ID (address ID) of the household assigned to each parcel is shown inside the house parcel (the map in right side). The ID is used to link household census and disease surveillance data.

The georeferenced image and the household parcel maps were used during a ground survey. The survey team consisted of civil engineers skilled in drawing household parcels and other field staff who conducted the census survey. The ground survey included verification of the size and orientation of household structures, resketching of the structures where needed, and locating specific household parcels so that they could be given their unique address identification number (ID) which was created during census survey (Figure 4). The address ID of each household was marked on the walls or doors at the time of census survey. Each time, the ground survey was started from a known location on the image, the household address ID was verified, and the IDs were marked on the hard copy map. Incorrect sizes or orientations of the digitized house parcels were also corrected during the ground survey.

After completing the ground surveys, the maps were updated using AutoCAD, and the address IDs were added to household parcels in the database. Finally, the household parcel AutoCAD files were imported as polygons into the ArcGIS software package (ESRI Inc., USA). The process included several checks for missing households, duplicate address IDs, and misplacement of address IDs and data were corrected when an error was found. The corrected data were validated by randomly selecting several household parcels (about 2%) from different zones of the study area and verifying their position on ground. At this stage, we observed no discrepancies in the data between ground verification and the satellite based maps suggesting that the household level GIS database is highly accurate.

## Implementation of the health GIS (HGIS) study

**Figure 5:**
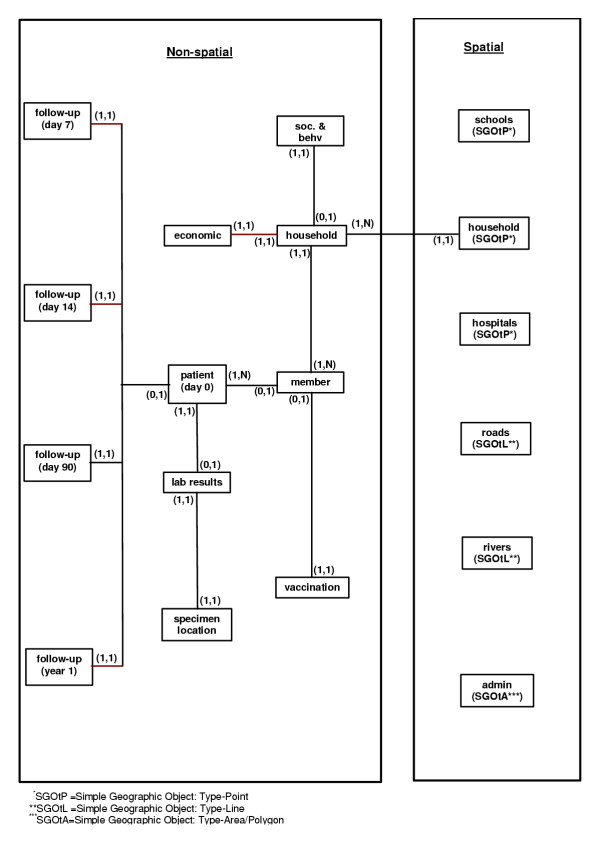
**Entity relationship of the health study GIS database, Karachi, Pakistan. **Inter-relationship between spatial and non-spatial database and intra-relationship of the database tables are shown here for the GIS-based health study research project. The descriptions of the entity relationship are descried in the texts.

The HGIS database is composed of spatial and non-spatial components (Figure 5). The spatial component consists of geographic features of households, roads, rivers, prominent places (e.g., hospitals), schools, and administrative boundaries. Each type of geographic feature was drawn in a single map layer. For instance, although both hospitals and schools were spatially referenced by points, we created two map layers for these two types of geographic features. The non-spatial component of the database consists of household-level data that include household socioeconomic status and individual-level data such as vaccination and disease history. The database relationships for patients with target diseases are shown in Figure 5.

Entity relationships between data tables are shown as lines, and logical relationships between entities have parentheses around them (Figure 5). In the logical relationship (1,N), "1" indicates each entity should be linked to an entity on the other end, and "N" indicates multiple entities can be linked to an entity at the other end. Similarly, the "0" in (0,1) indicates not all entities will be linked to an entity at the other end. The "1" of the relationship indicates not more than one entity will link to an entity at the other end. For example, the relationship of "member" towards "patient" is shown as (0,1). Here "0" indicates not all records in "member" are to be linked in "patient," and "1" indicates not more than one record of the "member" can be linked to a record in "patient". Similarly, "1" in the relationship (1,N) of "patient" towards "member" indicates all records in "patient" should be linked to "member', and "N" indicates multiple records in "patient" can be linked to a record in the "member".

## Conclusion

In this paper, we have outlined methodological issues involved in the construction of a household GIS database using satellite-based technology in a situation where the GPS was not appropriate. To our knowledge, this approach has never been reported, but may offer greater value in constructing household GIS databases compared to that based on GPS. Our household GIS offers size and orientation of individual houses in dense urban environment. Such database can be instrumental in health and disease studies because they facilitate the integration of socioecological and environmental factors that may influence health. Future health studies may benefit by using satellite-based technology to construct household GIS databases.
